# Physical Characterization and Effect of Effective Surface Area on the Sensing Properties of Tin Dioxide Thin Solid Films in a Propane Atmosphere

**DOI:** 10.3390/s140100403

**Published:** 2013-12-27

**Authors:** Heberto Gómez-Pozos, José Luis González-Vidal, Gonzalo Alberto Torres, María de la Luz Olvera, Luis Castañeda

**Affiliations:** 1 Área Académica de Computación, ICBI, Universidad Autónoma del Estado de Hidalgo, Mineral de la Reforma, 56092 Hidalgo, Mexico; E-Mails: gpozos@uaeh.edu.mx (H.G.-P.); jlvidal@uaeh.edu.mx (J.L.G.-V.); torres@uaeh.edu.mx (G.A.T.); 2 Departamento de Ingeniería Eléctrica-SEES, Centro de Investigación y de Estudios Avanzados del Instituto Politécnico Nacional, CINVESTAV-IPN, Apartado postal 14740, México D.F. 07000, Mexico; E-Mail: molvera@cinvestav.mx; 3 Escuela Superior de Ingeniería Mecánica y Eléctrica Unidad Ticomán, Instituto Politécnico Nacional, 07340 México, D.F., Mexico

**Keywords:** gas sensors, SnO_2_, thin solid films

## Abstract

The physical properties and the effect of effective surface area (ESA) on the sensing properties of tin dioxide [SnO_2_] thin films in air and propane [C_3_H_8_] atmosphere as a function of operating temperature and gas concentration have been studied in this paper. SnO_2_ thin films with different estimated thicknesses (50, 100 and 200 nm) were deposited on glass substrates by the chemical spray technique. Besides, they were prepared at two different deposition temperatures (400 and 475 °C). Tin chloride [SnCl_4_ · 5H_2_O] with 0.2 M concentration value and ethanol [C_2_H_6_O] were used as tin precursor and solvent, respectively. The morphological, and structural properties of the as-prepared films were analyzed by AFM and XRD, respectively. Gas sensing characteristics of SnO_2_ thin solid films were measured at operating temperatures of 22, 100, 200, and 300 °C, and at propane concentration levels (0, 5, 50, 100, 200, 300, 400, and 500 ppm). ESA values were calculated for each sample. It was found that the ESA increased with the increasing thickness of the films. The results demonstrated the importance of the achieving of a large effective surface area for improving gas sensing performance. SnO_2_ thin films deposited by spray chemical were chosen to study the ESA effect on gas sensing properties because their very rough surfaces were appropriate for this application.

## 1. Introduction

Gas sensors based on semiconductor oxides play an important role in the detection of toxic gases such as carbon monoxide [CO] [1], nitrogen oxide [NO_x_] [2], carbon dioxide [CO_2_] [3], hydrogen sulfide [H_2_S] [4], sulfur dioxide [SO_2_] [5], and some inflammable gases, for example hydrogen [H_2_] [6], methane [CH_4_] [7], and propane [C_3_H_8_] [8], among others. The SnO_2_-based gas sensor is an n-type semiconductor oxide due to the non-stoichiometry associated with the oxygen vacancies and/or tin excesses, which act as donor states providing conduction electrons. Its electrical surface resistance is highly influenced by the atmosphere since this surface and inter-grains chemisorb oxygen from the air at room temperature. Thus, when the surface is placed under an oxidizing atmosphere, the surface states physisorbed atomic and molecular ox0ygen; those captured conduction electrons produce a blending of the energy bands in this surface region, which leads to a surface electrical resistance increase [9]. On the other hand in the presence of a reducing gas, the electrons trapped by the oxygen species are released due to reaction between the reducing molecules and the physisorbed oxygen species, resulting from this fact a decrease of the surface electrical resistance [10]. Different physical and chemical deposition techniques have been utilized for depositing SnO_2_ thin films. Among the physical techniques are sputtering [11], chemical vapor deposition [12], and evaporation [13], among others whereas sol-gel [14] and spray pyrolysis [15] are the most used chemical techniques used to deposit SnO_2_ in thin films. This material is widely used for manufacturing commercial gas sensing devices [10].

The main goal of this research was to present some results of our explorative investigations on the physical characteristics of chemically sprayed SnO_2_ thin films and the effect of changing the ESA of these thin solid films on the sensing properties in a controlled propane atmosphere. The ESA of SnO_2_ samples were varied using various film thicknesses and digital image processing was used to calculate ESA values. Furthermore, the influence of deposition conditions and ESA on the gas sensing properties of SnO_2_ thin films were investigated and discussed. The SnO_2_ films with 200 nm of thickness exhibited the highest sensitivities, around 0.7 for 300 ppm of propane, measured at a 300 °C.

## 2. Experimental Procedure

### 2.1. Tin Dioxide Thin Solid Films Preparation

The samples were prepared using a 0.2 M starting solution of stannous chloride [SnCl_2_ · 5H_2_O] (98% from Alfa Aesar, Ward Hill, MA, USA) dissolved in ethanol [C_2_H_6_O] (98%, from J.T. Baker, Naucalpan, Edo. Mex., Mexico). The SnO_2_ thin solid films were deposited on clean soda-lime glass substrates (2.5 cm × 2.5 cm × 0.5 cm) by chemical spray technique. Spray pyrolysis involves the application of a fine mist of very small droplets containing the desired reactants onto hot substrates. The most critical operations of the spray pyrolysis technique were the preparation of fine uniform droplets and the controlled thermal decomposition of these droplets. The following cleaning procedure was used: (i) a five minutes wash in trichloroethylene [C_2_HCl_3_] (98%, from J.T. Baker) to degrease the substrate; (ii) followed by five minutes washing in acetone [CH_3_COCH_3_] (98%, from J.T. Baker); (iii) then five minutes washing in methyl alcohol [CH_3_OH] (98%, from J.T. Baker), and finally; (iv) drying under a nitrogen flow [N_2_] (99%, from PRAXAIR, Distrito Federal Mexico). All washing steps were carried out in an ultrasonic water bath. The substrate temperature was varied from 400 to 475 °C, with an accuracy of ±1 °C. The solution and carrier flow rates were held constant at 12 mL·min^−1^, and 10 L·min^−1^, respectively. In order to analyze the effect of the film thickness on the sensitivity properties, samples with three different thicknesses were deposited. The film thickness could be roughly estimated by measuring the deposition time. The process was stopped when the deposition times were 1.0, 2.0 and 5.0 min, which was approximately equivalent to a thickness of 50, 100 and 200 nm, respectively.

### 2.2. Thin Solid Films Characterization

A KLA Tencor P-15 profilmeter (Filmetrics, San Diego, CA, USA), with a resolution of 0.1 nm, was used to measure the thicknesses. The micro-crystalline structure was determined using the X-ray diffraction patterns which was obtained from a Siemens D5000-diffractometer (Siemens, Munich, Germany) by the θ-2θ technique, using Cu-K_α1_ (λ = 0.15405 nm) radiation. The surface morphology was analyzed by atomic force microscopy (AFM) on a Veeco-Autoprobe CP Research apparatus (Veeco, Plainview, NY, USA) with a force of 12 N and a gain of 0.05 a. u. on the contact mode. On the other hand, it is necessary to point out that noise was often present in the AFM images and could be caused by a number of factors, such as the environment's noise level (to mention some: acoustic, vibration and electromagnetic noise), so in some cases noise in the images had to be cleaned using suitable filters.

### 2.3. Sensing Properties

The sensing properties were determined by *in-situ* electric resistance measurements (Keithley 200, Keithley Instruments, Inc, Cleveland, OH, USA), by mean of two linear ohmic contacts, which were manufactured onto the films surface using a silver conductive adhesive (Alfa-Aesar). The samples were placed in a vacuum-chamber containing propane gas and they were heated by a heater that had a copper wire electrical resistance on the bottom. The operational temperature was electronically controlled by a J-thermocouple for four different fixed values (22, 100, 200, and 300 °C). The gas concentration was electronically controlled by a Leybold-T20 vacuum meter (Oerlikon Leybold Vacuum, Cologne, Germany). The propane concentration was varied in a range from 0 to 500 ppm. The sensitivity values *S*, were calculated using the following mathematical expression [16]:

(1) $$S=\frac{G_{G}+G_{O}}{G_{O}}$$

where *G_O_* was the electrical conductance of the sensor measured in air as reference gas, and *G_G_* was the electrical conductance in presence of the gas propane measured at specific conditions.

**Figure 1.**
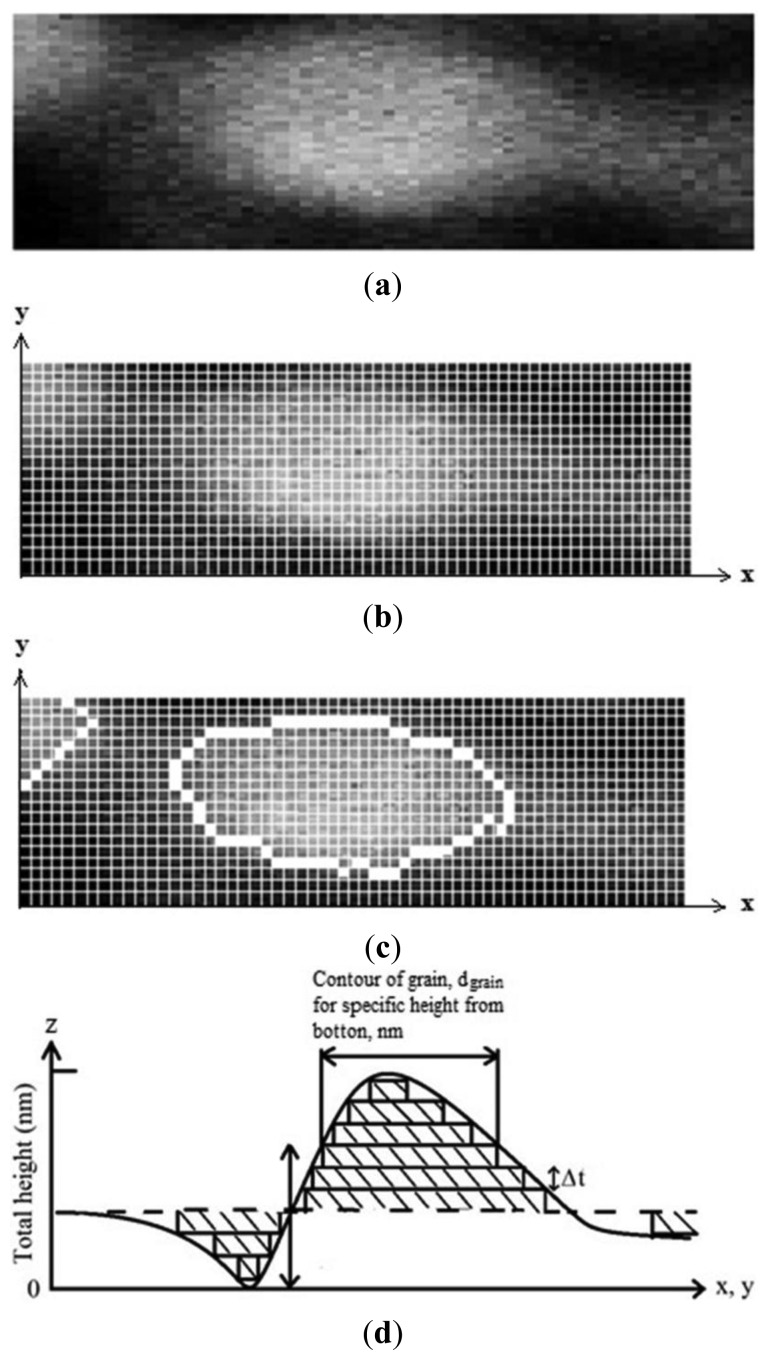
(**a**) Grain in 256 grayscale; (**b**) region (shaded) as it is transformed from discrete form and (**c**) then considered as a contour; (**d**) calculation of surface of a grain.

Either the ESA or the surface exposed to gas was calculated using a digital image processing technique. The process to obtain the effective area from AFM image can be described as follows: (i) the AFM images must be 256 grayscale, seeing the image from above, the top was the whitest area and the bottom was the darkest (Figure 1a shows only a grain); (ii) segmentation: in order to distinguish between the grains of interest and “the rest” or the background, segmentation techniques are used. In this work, a contour finding technique based on the Sobel gradient [17], combined with the Isodata thresholding algorithm [18] was used to find the pixels that belong to the borders of the grains (Figure 1b,c); (iii) calibration and contour measurements of grains, *d_grain_*. Dimensions of the images in x, y, and z coordinates (Figure 1b,c) were obtained from AFM measurements. Therefore, it was possible to know contour value of the grains giving numerical values or by calibrating to the pixel. If all pixels present at the contour of a grain (white squares, Figure 1c) are added and multiplied by the numerical value of the pixel, the whole contour value of grain must be obtained; (iv) the whole surface of a grain would be obtained as the sum of all grain contours, *d_grain_*; the computerized process began from bottom to top of grain for each increase of a proposed (*Δt*) width. If *Δt* → 0, the surface of grain must be more accurate measurement, (Equation (2)) as it was depicted by Figure 1d:

(2) $$\text{The whole surface of a grain}=\sum_{i=\text{bottom}}^{\text{top}}d_{{\text{grain}}_{i}}\Delta t$$

Finally, (v) the effective surface area (ESA) will be obtained as the summation of the surfaces of all grains in the sample. The ESA results for each image are given in Section 3.2.1.

## 3. Results and Discussion

### 3.1. Structure Properties

**Figure 2.**
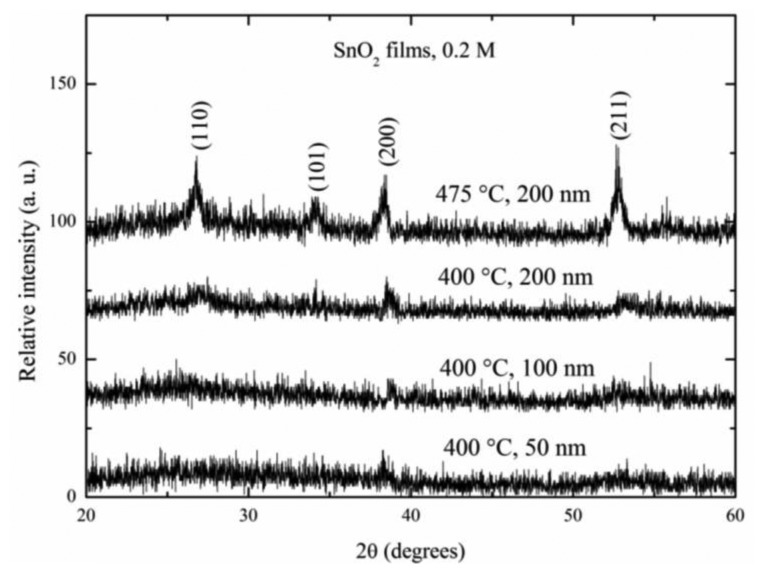
X-ray diffraction patterns of the SnO_2_ deposited at 400 and 475 °C with different thickness magnitude.

Figure 2 shows the X-ray diffraction patterns for SnO_2_ thin films deposited at 400 °C with different thicknesses: 50, 100 and 200 nm and for SnO_2_ films deposited at 475 °C with a film thickness of 200 nm.

The diffraction patterns were detected in a 2θ interval from 20 to 60°. The noisy-lines presented in all the spectra were a consequence of the small thickness. The existence of sharp peaks confirmed the films were polycrystalline. Films deposited at 400 °C did not show any significant peaks, irrespective of the thickness value. This very low intensity presented in the thinnest films was an expected result due to the small quantity of material compared to the thicker films. However, the SnO_2_ film which was deposited at 475 °C with a film thickness of 200 nm showed the main four peaks in its pattern. It clearly presented the main well-positioned peaks according to the JCPDS tables, since they fitted well with the tetragonal crystal structure [19]. The prevalence of the (110), (101), (200) and (211) planes were typically observed in thick films deposited at 475 °C [20]. From these pattern was evident the importance of using high deposition temperatures in the obtaining of better crystalline structures, it meant, the crystallinity of the SnO_2_ films was improved by the increasing of the deposition temperature.

### 3.2. Morphological Properties

**Figure 3.**
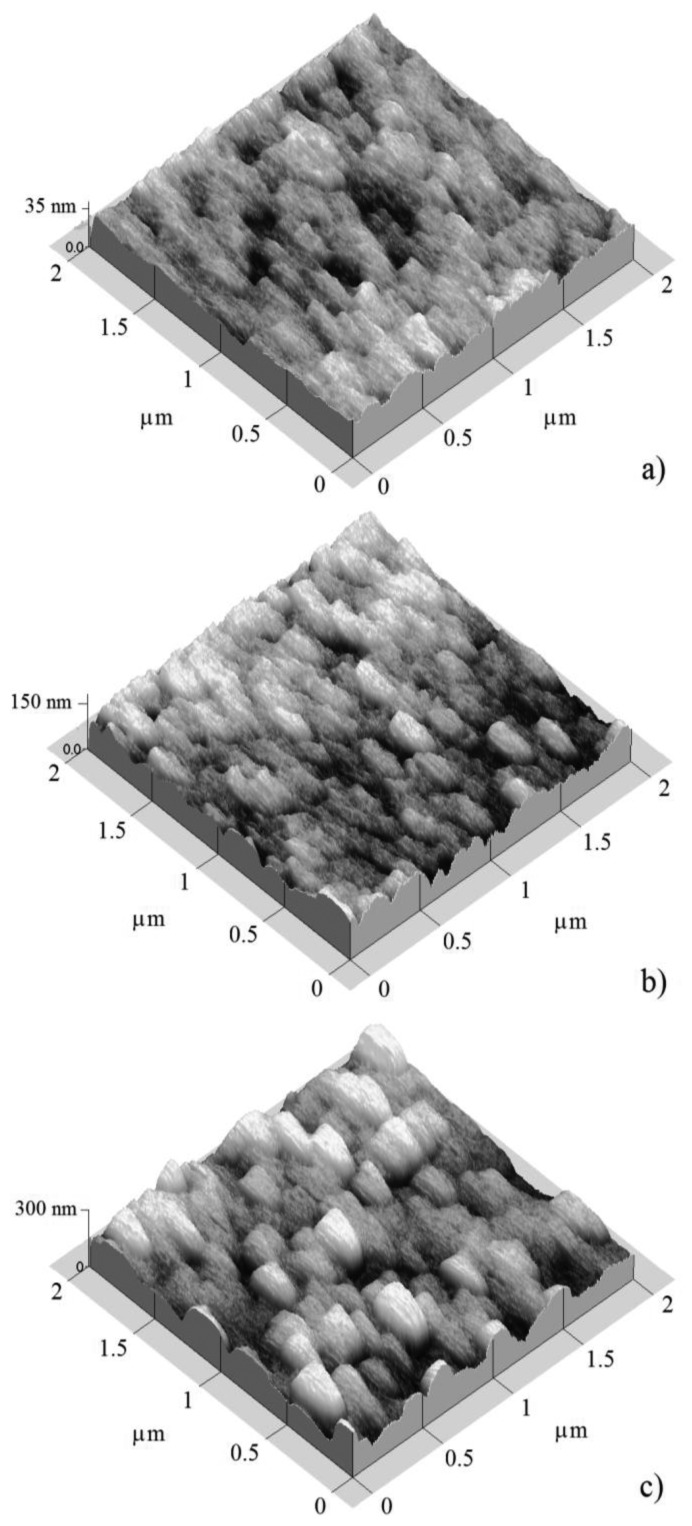
AFM images of SnO_2_ thin solid films deposited at 400 °C and three different thickness values: (**a**) 50; (**b**) 100; and (**c**) 200 nm.

**Figure 4.**
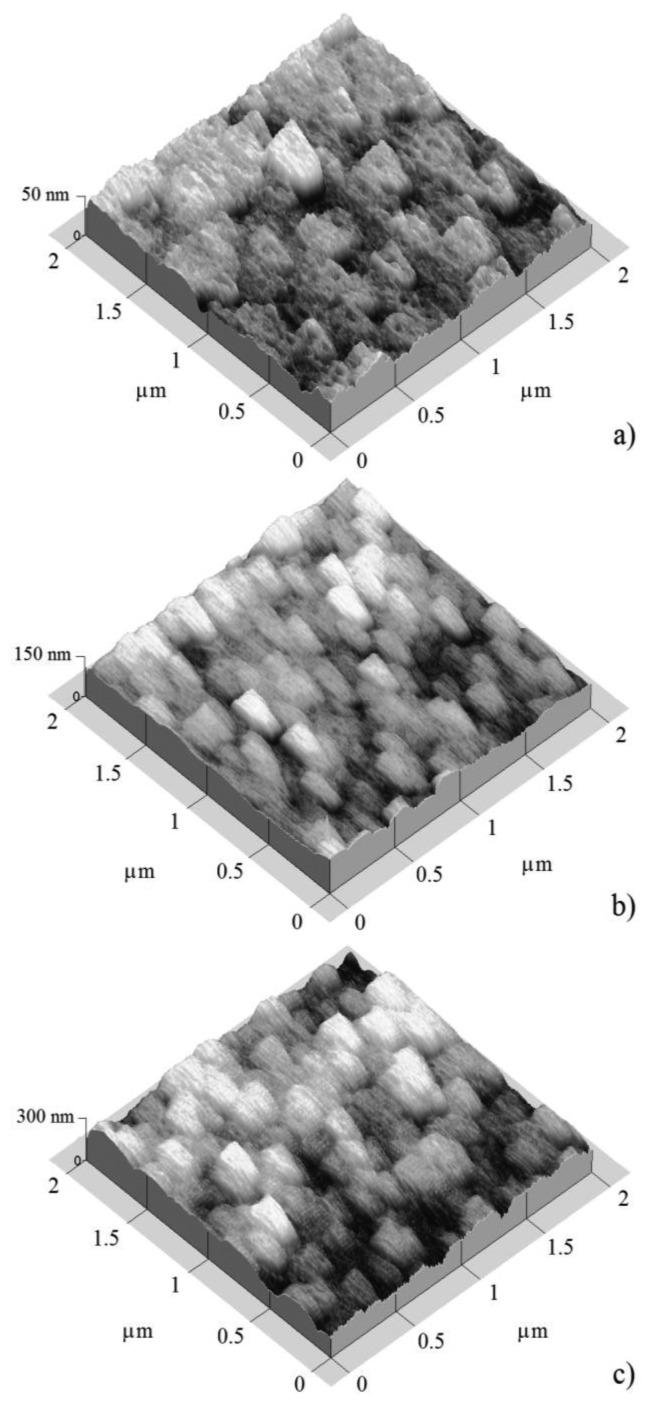
AFM images of SnO_2_ thin solid films deposited at 475 °C and three different thickness values: (**a**) 50; (**b**) 100; and (**c**) 200 nm.

Figures 3 and 4 show atomic force micrographs of SnO_2_ films deposited at 400 and 475 °C, respectively.

According to the micrographs, the film morphology showed a dependence on the estimated film thickness (50, 100, and 200 nm). SnO_2_ samples which were deposited at 400 °C with films thicknesses of 50, 100, and 200 nm are shown in Figures 3, respectively.

In general, the three micrographs showed a surface morphology consisting of grains with different grain size, or feature size, separated by empty spaces or grain boundaries. The grain size magnitudes depend slightly on the film thickness. Figure 3a shows the surface morphology of the 50 nm thick film. An irregular surface covered by round grains with an average diameter of 25 nm, and other elongated grains of 150 nm × 250 nm was observed. The largest grains seemed to be made up of conglomerates of smaller grains. Films with intermediate thickness (100 nm) are shown in Figure 3b. This presented a surface covered by elongated grains of 150 nm × 300 nm size. Hence, the film was more compact.

The thin solid films with thickness of 50 and 100 nm presented a closely similar morphology, appearing as grains with elongate-like shapes, of the order 150 nm × 300 nm, and composed by smaller round grains which were formed in both cases. Finally, in the case of the thickest film (200 nm, Figure 3c), the surface was only composed of elongated grains of 200 nm × 350 nm, so we can conclude that the film thickness contributed to the growth of the grains, although these large grains were the result of the conglomeration of smaller grains.

Thin solid films which were deposited at 475 °C (Figure 4) showed a closed similar surface covered by enlarged grains with a size higher than the deposited films at 400 °C, at least thickness for films thickness of 50 and 100 nm. The thinnest sample (50 nm, Figure 4a) showed elongated grains of 200 nm × 250 nm. In the case of the film with thickness of 100 nm, (Figure 4b) the grain shape was similar to that of the film with a thickness of 50 nm. For both films with thicknesses of 100 and 200 nm, the grains average size was closely similar. Finally in the case of the thickest 200 nm film, the surface seemed to be covered by two-form grains, round and elongated grains, and the grain size was increased slightly (Figure 4c).

#### 3.2.1. Effective Surface Area (ESA) of Tin Dioxide [SnO_2_] Thin Solid Films

ESA of these images was obtained in according to the processes described in the Experimental section. Table 1 shows the measured film thickness, total height, and ESA as a function of estimated SnO_2_ thin solid film thickness deposited at temperatures of 400 and 475 °C, the images of all of which were shown have a 2.0 × 2.0 μm^2^ area (Figures 3 and 4). The data in the Table shows that the measured film thickness, total height, and ESA increased with the increasing estimated film thickness. ESA of these images was nearly twice as much as areas of shown images (2.0 × 2.0 μm^2^). For SnO_2_ thin solid film at 400 °C, the ESA was much bigger than the SnO_2_ sample deposited at 470 °C. Therefore, the sample which was deposited at 400 °C had more contact area for detecting gases. The atoms had a high mobility to accommodate into the crystal lattice. Thus, the SnO_2_ film surface at 475 °C had a higher energy of formation, which resulted in a less rough and more compact and thinner surface.

### 3.3. Propane Sensing Properties

#### 3.3.1. Characterization of SnO_2_ Thin Solid Films Deposited at 400 °C

**Figure 5.**
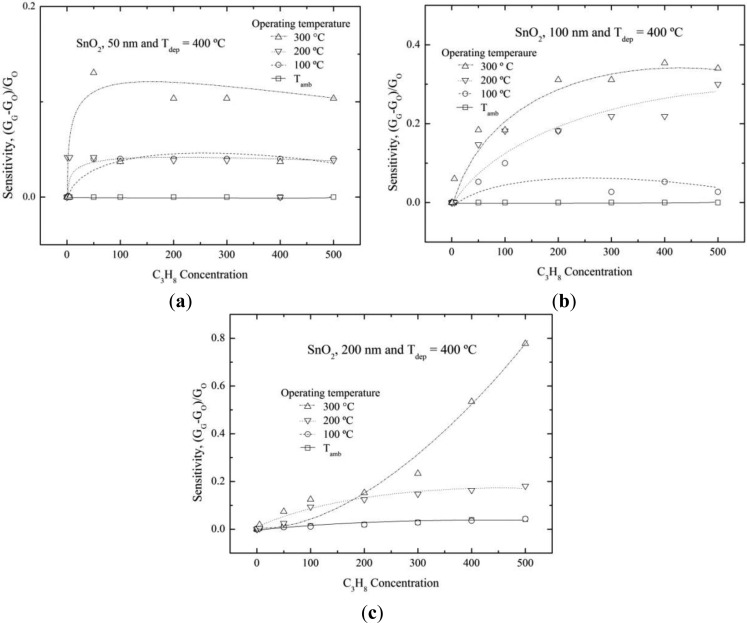
Sensitivity as a function of propane concentration and different operation temperature for SnO_2_ thin films deposited at 400 °C, (**a**) 50; (**b**) 100; and (**c**) 200 nm. Solid, dash, dotted and dash-dotted lines are guides to the eye.

**Figure 6.**
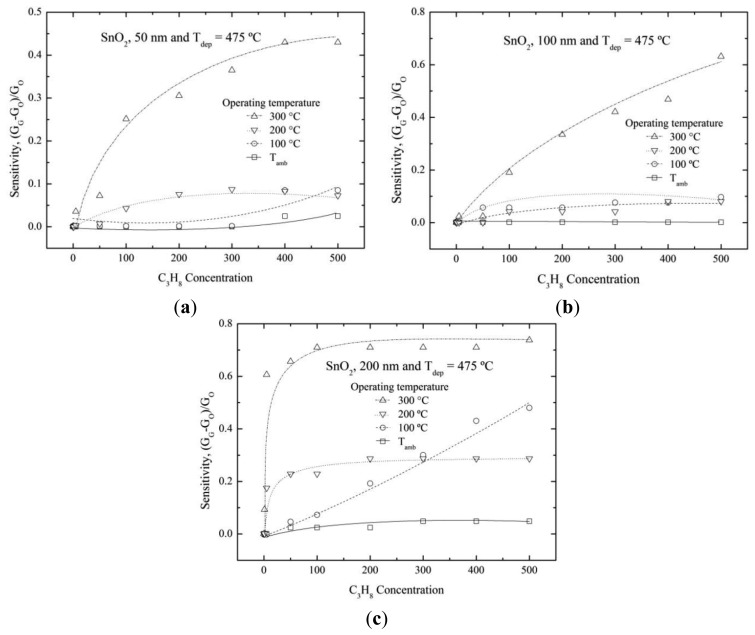
Sensitivity as a function of propane concentration and different operation temperature for SnO_2_ thin solid films deposited at 475 °C, (**a**) 50; (**b**) 100; and (**c**) 200 nm. Solid, dash, dotted and dash-dotted lines are guide to the eye.

The sensitivity values of SnO_2_ thin film sensors at different operational temperatures and C_3_H_8_ concentrations are shown in Figures 5 and 6. The sensitivity values were estimated by the formula which was given in the Experimental section. The surface electrical resistance was monitored in a hermetic chamber at different propane concentrations and operational temperatures. The sensitivity values varied with both propane concentration and operational temperature.

Figure 5a shows the sensitivity variation of the 50 nm thick SnO_2_ films. Films presented very low sensitivity magnitudes (∼0.1) at 300 °C. Furthermore, it was saturated at low propane concentrations (between 5 and 50 ppm). This behavior can be explained by the interaction of propane particles with the desorbed oxygen species as the operating temperature increased. Nonetheless, there were not more oxygen species to interact with propane species on the film surface above 50 ppm propane concentration. Consequently, no more electrons could be released since there were not enough oxygen species that have been desorbed by its chemical and electronic interaction with the propane. It was important to clear that all the measurements reported in the graph were made on the same sample; this fact showed the high recuperation properties of the films.

Figure 5b shows the concentrated propane dependence of sensitivity for 100 nm SnO_2_ film. The film had higher sensitivity values (∼0.35) than in the previous case (Figure 5a). However in this case, the sensitivity was not saturated for low propane concentration.

Figure 5c shows the sensitivity variation of the thickest (200 nm) SnO_2_ film. This film presented the highest sensitivity values (∼0.80). As a general trend, we observed that the sensitivity increased as the film thickness increased, showing that this was a surface effect. In fact, the atomic disorder on the surface decreased as the film thickness increased. The surface energetic states were more available for thick film thicknesses since the film was better formed and therefore there were more interactions between the SnO_2_ surface film and the propane molecules. In general, the sensitivity was increased as the propane concentration was increased from 50 to 500 ppm. However, the sensitivity was decreased when the propane gas was removed from the chamber, indicating that the films had a high-quality response for different propane concentrations.

#### 3.3.2. Characterization of SnO_2_ Thin Solid Films Deposited at 475 °C

Figure 6a shows the sensitivity variation as a function of the propane concentration for SnO_2_ film with 50 nm thickness and deposited at 475 °C. An increase in the sensitivity was observed as the operational temperature was increased at 300 °C, reaching a maximum value of 0.4. It was higher than films deposited at 400 °C. Then, films with 50 nm thickness and deposited at 475 °C offered slightly better results. The 100 nm SnO_2_ thin film showed high sensitivities at 300 °C and at propane concentrations higher than 100 ppm (Figure 6b). The maximum sensitivity value about of ∼0.6 was obtained for 500 ppm. The sensitivity behavior of the thickest SnO_2_ films (200 nm) is shown in Figure 6c. The films presented the highest sensitivity value, ∼0.7. It could be inferred from this result that the sensitivity was favored by thick deposited films. The higher deposition temperatures improved the sensing properties too.

### 3.4. Results of the Effective Surface Area (ESA) on Sensing Properties

**Figure 7.**
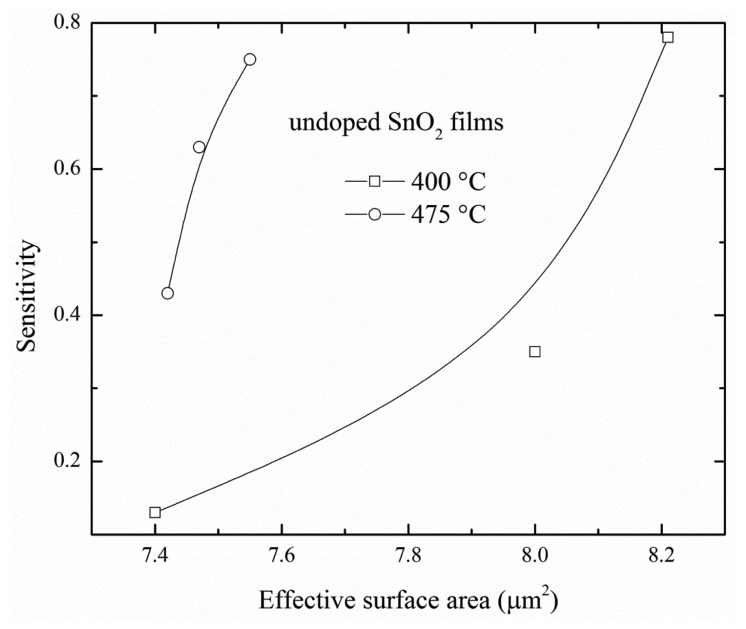
Dependence of sensitivity of SnO_2_ films on effective surface area.

The Figure 7 shows the sensitivity according to ESA of SnO_2_ films at 400 and 475 °C. The figure revealed that the sensitivity was increased as the ESA increased; this result was associated with a bigger contact area for detecting gas. It could be seen that for film thicknesses higher than 270 nm the sensitivity was not saturated (Figure 7). Therefore, an increase in the sensitivity could be obtained by a further increase in the film thickness, the sensitivity would reach the value maximum for a given film thickness, by way of explanation, the largest ESA that the films could reach.

The sensitivity has been studied as a function of the deposition parameters, such as deposition temperature, type of catalysis, deposition techniques, solvents, staring solution, among other. Moreover, it could be expected that the sensitivities from both deposited SnO_2_ films would be different for similar ESA values. In this study, for an ESA less than 7.6 μm^2^, the sensitivity of SnO_2_ films deposited at 475 °C is larger than that of SnO_2_ films deposited at 400 °C due to the fact that the morphology of SnO_2_ films deposited at 475 °C is better than that of SnO_2_ films deposited at 400 °C. This indicated that there were a large amount of energy levels to catch C_3_H_8_ molecules on the surface, but for ESA values of more than 8.2 μm^2^, the sensitivity is similar for both films because there were more ESA in SnO_2_ films deposited at 400 °C compensating for the fewer energy levels on their surface.

## 4. Conclusions

The results obtained from a study on the physical properties and the effect of effective surface area on the sensing properties of chemically sprayed SnO_2_ thin films with different thicknesses (50, 100 and 200 nm) were presented in this work. The X-ray results revealed that the crystallinity of thin solid films was improved as the film thickness and deposition temperature was increased, this fact was convenient for the sensing properties. All the micrographs confirmed a surface morphology consisting of elongated and round grains with different grain sizes and separated by empty spaces. The large grains seemed to be conglomerates of smaller grains. The sensitivity was improved by increasing the operational temperature and propane concentration. The maximum sensitivities, around ∼0.7, were obtained for a 200 nm SnO_2_ thin film, measured at 300 °C in a propane atmosphere for films deposited at 400 °C and 475 °C. The effective surface areas were calculated by image processing techniques. For both deposited SnO_2_ films, the sensitivity was increased as the effective surface area increased. In this work, it was found that there was an almost linear relation between the sensitivity and effective surface area. The spray chemical technique produced surface roughness which increases the effective surface area.

## Figures and Tables

**Table 1.** Measured thickness film, total height and effective surface depending on film thickness of SnO_2_ at 400, and 475 °C.

**Deposition Temperature (°C)**	**Estimated Film Thickness (nm)**	**Measured Film Thickness (nm)**	**Total Height (nm)**	**Effective Surface Area (ESA) (μm^2^)**
400	50	30	16	7.41
	100	121	15	8.10
	200	270	24	8.21

475	50	36	17	7.42
	100	118	17	7.47
	200	249	24	7.55

## References

[1] Malyshev V.V., Pislyakov A.V. (2007). Investigation of gas-sensitivity of sensor structures to carbon monoxide in a wide range of temperature, concentration and humidity of gas medium. Sens. Actuators B Chem..

[2] Lee D.-S., Lim J.-W., Lee S.-M., Huh J.-S., Lee D.-D. (2000). Fabrication and characterization of micro-gas sensor for nitrogen oxides gas detection. Sens. Actuators B Chem..

[3] Desai R.R., Lakshminarayana D., Patel P.B., Panchal C.J. (2005). Indium sesquitelluride (In_2_Te_3_) thin film gas sensor for detection of carbon dioxide. Sens. Actuators B Chem..

[4] Vaishampayan M.V., Deshmukh R.G., Walke P., Mulla I.S. (2008). Fe-doped SnO_2_ nanomaterial: A low temperature hydrogen sulfide gas sensor. Mater. Chem. Phys..

[5] Das S., Chakraborty S., Parkash O., Kumar D., Bandyopadhyay S., Samudrala S.K., Sen A., Maiti H.S. (2008). Vanadium doped tin dioxide as a novel sulfur dioxide sensor. Talanta.

[6] Lupan O., Chai G., Chow L. (2008). Novel hydrogen gas sensor based on single ZnO nanorod. Microelectron. Eng..

[7] Angelis L., Riva R. (1995). Selectivity and stability of a tin oxide sensor for methane. Sens. Actuators B Chem..

[8] Carbajal-Franco G., Tiburcio-Silver A., Domínguez J.M., Sánchez-Juárez A. (2000). Thin film tin oxide-based propane gas sensors. Thin Solid Films.

[9] Mandelis A., Christofides C., Winefordner J.D. (1993). Physics, Chemistry, and Technology of Solid State Gas Sensor Devices. Chemical Analysis: A Series of Monographs on Analytical Chemistry and Its Applications.

[10] Korotchenko G., Brynzari V., Dmitriev S. (1999). SnO_2_ films for thin film gas sensor design. Mater. Sci. Eng. B Adv. Funct. Solid-State Mater..

[11] Shen Y., Yamazaki T., Liu Z., Jin C., Kikuta T., Nakatani N. (2008). Porous SnO_2_ sputtered films with high H_2_ sensitivity at low operation temperature. Thin Solid Films.

[12] Salehi A., Gholizade M. (2003). Gas-sensing properties of indium-doped SnO_2_ thin films with variations in indium concentration. Sens. Actuators B Chem..

[13] Brousse T., Schleich D.M. (1996). Sprayed and thermally evaporated SnO_2_ thin films for ethanol sensors. Sens. Actuators B Chem..

[14] Rella R., Serra A., Siciliano P., Vasanelli L., de G., Licciulli A. (1997). CO sensing properties of SnO_2_ thin films prepared by the sol-gel process. Thin Solid Films.

[15] Korotcenkov G., Brinzari V., Schwank J., DiBattista M., Vasiliev A. (2001). Peculiarities of SnO_2_ thin film deposition by spray pyrolysis for gas sensor application. Sens. Actuators B Chem..

[16] Sze S.M. (1993). Semiconductor Sensors.

[17] Gonzalez R., Woods R. (1992). Digital Image Processing.

[18] Velasco F.R.D. (1980). Thresholding using the ISODATA clustering algorithm. IEEE Trans. Syst. Man Cybern..

[19] (1967). Powder Diffraction File. Joint Committee on Powder Diffraction Standards.

[20] Korotcenkov G., Cornet A., Rossinyol E., Arbiol J., Brinzari V., Blinov Y. (2005). Faceting characterization of tin dioxide nanocrystals deposited by spray pyrolysis from stannic chloride water solution. Thin solid films.

